# Genetic diversities in wild and cultivated populations of the two closely-related medical plants species, *Tripterygium Wilfordii* and *T. Hypoglaucum* (Celastraceae)

**DOI:** 10.1186/s12870-024-04826-x

**Published:** 2024-03-16

**Authors:** Chao Liu, Jingyi Wang, Ya-Zhu Ko, Meng-Shin Shiao, Yiheng Wang, Jiahui Sun, Qingjun Yuan, Lisong Wang, Yu-Chung Chiang, Lanping Guo

**Affiliations:** 1 State Key Laboratory Breeding Base of Dao-di Herbs, National Resource Center for Chinese Materia Medica, China Academy of Chinese Medical Sciences, Beijing, 100700 China; 2 Department of Biological Sciences, National Sun Yat-sen University, Kaohsiung City, Taiwan; 3 Research Center, Faculty of Medicine, Ramathibodi Hospital, Mahidol University, Bangkok, 10400 Thailand; 4 Lushan Botanical Garden, Chinese Academy of Sciences, Jiujiang, Jiangxi 332900 China; 5 Department of Biomedical Science and Environmental Biology, Kaohsiung Medical University, Kaohsiung City, Taiwan; 6 The Multidisciplinary and Data Science Research Center(MDSRC), National Sun Yat-sen University, Kaohsiung, 804 Taiwan

**Keywords:** DNA sequence, Phylogeography, Population genetics, *Tripterygium*, Traditional chinese medicine

## Abstract

**Background:**

The sustainable supply of medicinal plants is important, and cultivating and domesticating them has been suggested as an optimal strategy. However, this can lead to a loss of genetic diversity. *Tripterygium wilfordii* Hook. f. is a medicinal plant commonly used in traditional Chinese medicine, but its wild populations are dwindling due to excessive harvesting. To protect the species and meet the increasing demand, it is urgent to cultivate it on a large scale. However, distinguishing between *T. wilfordii* and *T. hypoglaucum*, two similar species with different medicinal properties, is challenging. Therefore, it is crucial to understand the genetic diversity and population structure of these species for their sustainable utilization.

**Results:**

In this study, we investigated the genetic diversity and population structure of the two traditional medicinal semiwoody vines plant species, *Tripterygium wilfordii* and *T. hypoglaucum*, including wild and cultivated populations using chloroplast DNA (cpDNA) sequences and microsatellite loci. Our results indicated that the two species maintain a high level of genetic divergence, indicating possible genetic bases for the different contents of bioactive compounds of the two species. *T. wilfordii* showed lower genetic diversity and less subdivided population structures of both markers than *T. hypoglaucum.* The potential factors in shaping these interesting differences might be differentiated pollen-to-seed migration rates, interbreeding, and history of population divergence. Analyses of cpDNA and microsatellite loci supported that the two species are genetically distinct entities. In addition, a significant reduction of genetic diversity was observed for cultivated populations of the two species, which mainly resulted from the small initial population size and propagated vegetative practice during their cultivation.

**Conclusion:**

Our findings indicate significant genetic divergence between *T. wilfordii* and *T. hypoglaucum*. The genetic diversity and population structure analyses provide important insights into the sustainable cultivation and utilization of these medicinal plants. Accurate identification and conservation efforts are necessary for both species to ensure the safety and effectiveness of crude drug use. Our study also highlighted the importance of combined analyses of different DNA markers in addressing population genetics of medicinal plants because of the contrasts of inheritance and rates of gene flow. Large-scale cultivation programs should consider preserving genetic diversity to enhance the long-term sustainability of *T. wilfordii* and *T. hypoglaucum*. Our study proposed that some populations showed higher genetic diversity and distinctness, which can be considered with priority for conservation and as the sources for future breeding and genetic improvement.

**Supplementary Information:**

The online version contains supplementary material available at 10.1186/s12870-024-04826-x.

## Background

Medicinal plants play a central role in the health of humanity, but over-exploitation on wild resources led to biodiversity loss and caused global concerns [1–3]. Cultivation and domestication have been suggested as an optimal strategy for sustainable supply of medicinal plants [4–6]. However, the process of cultivation and domestication in most medicinal plants involves artificial selection and genetic bottleneck, which can lead to a serious loss of the genetic diversity found within the wild populations [7–9]. The loss of genetic diversity may have several severe impacts: it may lead to (1) decline of plasticity of secondary metabolism, which is the major source of medicinal compounds [10, 11], (2) progressive narrowing of the genetic diversity as the source for artificial selection to improve the yield of medicinal compounds [12–14], (3) lacking evolutionary potential to adapt to changing environments [15–17], and (4) negative impact on sustainable utilization. In particular, some promising medicinal plants for new drug development are being over-harvested, and their genetic diversity is rapidly declining. In order to protect the wild resources of medicinal plants and meet the increasing demand of human beings, there is an urgent need to introduce these medicinal plants into large-scale cultivation. Investigating genetic diversity patterns in wild and cultivated populations of these medicinal plants can provide guidelines for future cultivation programs.

*Tripterygium wilfordii Hook. f.* is a deciduous subshrub or scandent semiwoody vine of the family Celastraceae, the root and rhizome of which is a very important *materia medica* in traditional Chinese medicine. During the last two decades, *T. wilfordii* has attracted much attention due to new discoveries about its pharmacological activities, such as immunosuppressive, anti-inflammatory, anticancer and anti-HIV activity [18–22]. Triptolide is the major bioactive component of *T. wilfordii* [23], which is a primary diterpene triepoxide with immunosuppressive and anti-inflammatory pharmacological effects [24]. Analogs of triptolide have been shown to be effective in treating different diseases: Minnelide is a water-soluble triptolide analog, which has been shown to be highly effective in reducing pancreatic tumor and small cell lung carcinoma tumor growth and spread in animal models [25–27]; (5R)-5-Hydroxytriptolide (LLDT-8) is a new and optimized triptolide analog with lower cytotoxicity and relatively higher immunosuppressive activity [28]. These new pharmacological effects rapidly increased the demand for *T. wilfordii*. At present, the source of *T. wilfordii* medicinal materials mainly depends on the collection of wild resources, which results in the rapid decline in the sizes of the natural populations of *T. wilfordii* [22]. Thus, it is urgent to cultivate *T. wilfordii* artificially on a large scale.

In China, there are three species of *Tripterygium* Hook. f. in Flora Republicae Popularis Sinicae: *T. wilfordii* Hook. f., *T. hypoglaucum* (H. Lév.) Hutch, and *T. regelii* Sprague et Takeda [29]. The wild populations of *Tripterygium* species are restricted distribution: The wild *T. wilfordii* and *T. hypoglaucum* are native to eastern and southern China, *and T. regelii is mainly distributed in northeast* China. Wild *T. wilfordii* and *T. hypoglaucum* are plants growing naturally without human intervention. The wild *T. wilfordii* can be found in various habitats such as forests, hills, mountains, alpine regions, and other diverse terrain conditions [30]. Conversely, cultivated *T. wilfordii* and *T. hypoglaucum* are grown in controlled environments for selective planting and breeding. The cultivation of *T. wilfordii* mainly involves wild transplants, seedling cultivation, and cutting propagation, while cultivated *T. hypoglaucum* primarily relies on cutting propagation. *T. hypoglaucum* is also an important medicinal plant in traditional Chinese medicine, but its pharmacological effect differs from that of *T. wilfordii*. *Tripterygium wilfordii* and *T. hypoglaucum* have distinct chemical compositions. *Tripterygium hypoglaucum* consists of various unique secondary metabolites, and the content of its compounds differs from *T. wilfordii*. In particular, the sesquiterpene alkaloid content noticeably varies between the two. Medicinally, *T. hypoglaucum* is believed to have fewer side effects and lower toxicity than *T. wilfordii* [18, 31]. However, there is no significant difference in morphological characteristics, geographical distribution and chemical composition between *T. wilfordii* and *T. hypoglaucum*, making identifying them difficult [18, 32, 33]. Therefore, some authors merged *T. wilfordii* and *T. hypoglaucum* into one species [22, 34]. *Tripterygium regelii* is an independent species due to significant differences from the other two species in morphological characteristics, geographical distribution and chemical composition [18, 22, 32–34]. In *Flora of China*, the three species of *Tripterygium* have been merged into one species [35]. However, *T. wilfordii* and *T. hypoglaucum* are two completely different types of materia medica in traditional Chinese medicine, the accurate identification of which is relevant to the safety of crude drug use and sustainable utilization of the two species.

Genetic markers with higher resolutions have been used independently to study further the divergence of *Triptergium* species including random amplified polymorphic DNA (RAPD) [36], nuclear ribosomal internal transcribed spacer (ITS) and 5 S rDNA [37], chloroplast DNA (*psb*A-*trn*H, *rpl*32-*trn*L, and *trn*L-*trn*F), DNA barcodes (ITS2, *psb*A-*trn*H, *mat*K, and *rbc*L) and simple-sequence repeats (also known as microsatellite markers, SSRs) [34, 38]. However, the relationship and genetic structure of *Triptergium* species are inconclusive from these studies due to limited samples or insufficient genetic markers. Furthermore, the evolutionary rate, inheritance and dispersion mechanisms of different genetic markers are different [39, 40], which might be another reason for inconsistent results.

Chloroplast DNA (cpDNA) and microsatellite markers (SSRs) are commonly used genetic markers in studying population genetics. They have different mutation rates, inherited and dispersed mechanisms [39, 40]. Chloroplast DNA evolves relatively slowly and is maternal inheritance, reflecting the impacts of seed flow on genetic diversity. Microsatellite markers evolve relatively quickly and are biparental and codominant inheritance, reflecting the impacts of pollen flow on genetic diversity patterns [41, 42]. The cpDNA and SSRs markers have been successfully employed to detect genetic diversities in numerous plant species [43–49]. Nevertheless, few studies use both markers together to focus on the population genetics of medicinal plants. Here, we utilized two cpDNA regions and ten microsatellite loci to investigate the genetic diversity and genetic structure of *T. wilfordii* and *T. hypoglaucum* to (1) elucidate the phylogenetic relationships between *T. wilfordii* and *T. hypoglaucum*; (2) evaluate the level of genetic diversity and population structure of the two species; (3) compare the amount of genetic variation in cultivated and wild populations of the two species and provide sustainable utilization strategy.

## Results

### Higher number of haplotypes was observed in *T. hypoglaucum* while higher genetic diversity was observed *in T. wilfordii* using cpDNA sequences

**Fig. 1:**
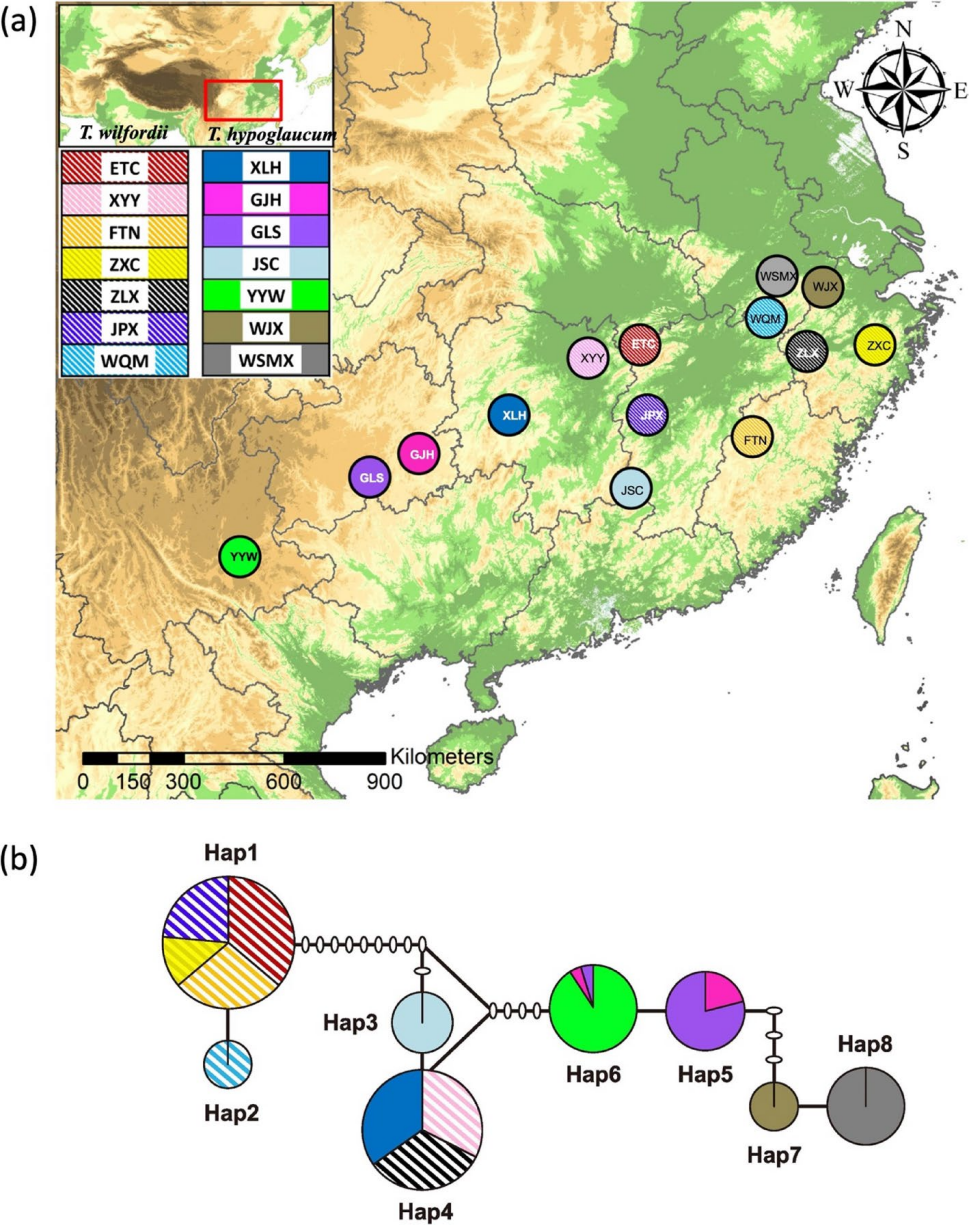
**a** Geographic distribution of *Tripterygium wilfordii* and* Tripterygium hypoglaucum* populations. **b** Median-joining networks of eight cpDNA haplotypes (Hap1-8) identified in this study. The sizes of the circles are proportional to the overall frequency of the haplotypes in the entire sample of all population. The white oval are the missing or inferred haplotypes and each line represents one mutational step

We collected samples from 14 populations, 6 wild and 1 cultivated for both *T. wilfordii* and *T. hypoglaucum* (Table 1). Sequences of cpDNA (*psb*A*-trn*H and *trn*L*-trn*F) were obtained from a total of 179 individuals and submitted to GenBank (GenBank accession numbers: PP128529-PP128707 for *psb*A*-trn*H; PP128708-PP128886 for *trn*L*-trn*F). A total of 8 haplotypes (Hap1-8) were identified using combined sequences of the two cpDNA regions, where a total of 18 polymorphic sites were identified. Among those, one haplotype (Hap4) was shared in two populations of *T. wilfordii* (XYY and ZLX) and in one population of *T. hypoglaucum* (XLH), which indicates possible gene flows between the two species either naturally or artificially. Other than the shared haplotypes, two haplotypes (Hap1 and 2) were identified specifically in *T. wilfordii*, and five haplotypes (Hap3, 5, 6, 7 and 8) were identified specifically in *T. hypoglaucum* (Table 1; Fig. 1).

**Table 1:** Sampling information and haplotypes of *Tripterygium wilfordii* (*Tw*) and *Tripterygium hypoglaucum* (*Th*) for chloroplast DNA (cpDNA) and microsatellite loci (SSRs) analyses

Taxon	Location	Code	State	Longitude (E)	Latitude (N)	Sample size		cpDNA Haplotype (Sample numbers)
						SSR	cpDNA	
*Tripterygium wilfordii*	Tongcheng County, Hubei	ETC	Wild	113°53’43.880”	29°16’06.640”	20	17	Hap1 (17)
	Yueyang City, Hunan	XYY	Wild	113°18’54.690”	29°02’30.920”	20	13	Hap4 (13)
	Taining County, Fujian	FTN	Wild	117°21’35.690”	27°45’16.990”	20	13	Hap1 (13)
	Xinchang County, Zhejiang	ZXC	Cultivar	120°56’32.606”	29°21’36.332”	9	6	Hap1 (6)
	Lanxi City, Zhejiang	ZLX	Wild	119°45’16.600”	29°20’54.300”	22	13	Hap4 (13)
	Pingxiang City, Jiangxi	JPX	Wild	113°55’58.740”	27°42’55.147”	21	11	Hap1 (11)
	Qimen County, Anhui	WQM	Wild	117°23’19.250”	29°56’51.410”	10	9	Hap2 (9)
*Tripterygium hypoglaucum*	Longhui County, Hunan	XLH	Wild	110°44’40.730”	27°32’03.010”	22	14	Hap4 (14)
	Jianhe County, Guizhou	GJH	Cultivar	108°34’07.170”	26°39’27.020”	9	5	Hap5 (4), Hap6 (1)
	Leishan County, Guizhou	GLS	Wild	108°10’57.394”	26°22’36.866”	21	16	Hap5 (15), Hap6 (1)
	Suichuan County, Jiangxi	JSC	Wild	114°00’24.939”	26°14’33.489”	20	13	Hap3(13)
	Yuxi City, Yunnan	YYW	Wild	102°10’17.350”	23°56’06.560”	21	21	Hap6 (21)
	Jixi County, Anhui	WJX	Wild	118°45’19.500”	30°05’40.500”	9	9	Hap7 (9)
	Huangshan City, Anhui	WSMX	Wild	118°14’21.000”	30°09’02.100”	20	19	Hap8 (19)
Total						244	179	

The abbreviations of code denote sample location. Professor Lisong Wang undertook the formal identification of the voucher specimens, which were deposited in herbaria of Institute of Chinese Materia Medica (CMMI), China Academy of Chinese Medical Sciences

Overall, polymorphic sites (*P*), nucleotide diversity ($\pi$), nucleotide polymorphism ($\theta w$), and haplotype diversity (*Hd*) were 18, 0.00650, 0.00264, and 0.836, respectively, from all the 179 individuals (Table 2). *T. wilfordii* exhibited more polymorphic sites, higher nucleotide diversity and nucleotide polymorphism (*P* = 12, $\pi$= 0.00424, $\theta w=$0.00204,) than those of *T. hypoglaucum* (*P =* 11, $\pi$= 0.00374, $\theta w=$0.00181). However, *T. hypoglaucum* showed higher number of haplotypes and higher haplotype diversity (*Nh =* 6, *Hd =* 0.828) than those of *T. wilfordii* (*Nh =* 3, *Hd =* 0.566). In addition, the neutrality test (Tajima’s *D* and Fu and Li’s F*) for linkage disequilibrium showed significance (*P* < 0.02 or *P* < 0.001) in both species. The results of Tajima’s *D* and Fu and Li’s F* tests show the significant positive value indicates that *T. wilfordii* and *T. hypoglaucum* may have experienced the effects of population bottlenecks or balancing selection. Consistent with this, the dynamic analysis of microsatellites also shows a decline in the current effective population size. This consistent result reveals that these species are facing the pressure of reduced genetic diversity.

**Table 2:** Genetic diversity estimated from cpDNA for all populations of *Tripterygium wilfordii* and *Tripterygium hypoglaucum*

	*N*	*P*	*π*	*θw*	*Nh*	*Hd*	*D*	D*	F*
All species	179	18	0.00650	0.00264	8	0.836	3.89391***	1.68314*	3.04834**
*Tw*	82	12	0.00424	0.00204	3	0.566	2.95790**	1.47296*	2.36612**
*Th*	97	11	0.00374	0.00181	6	0.828	2.80140***	1.42315*	2.26723**

*N* sample size, *P* polymorphic sites, *π *nucleotide diversity, *θw *nucleotide polymorphism, *Nh* No. of haplotype, *Hd* Haplotype diversity, *D* Tajima’s *D*, D* Fu and Li’s D*, F* Fu and Li’s F*

Significance level: * *P* < 0.05, ** *P* < 0.02, *** *P* < 0.001

### Geographic distribution and genealogy of haplotypes demonstrated that the populations of the two species have different evolutionary relations

The geographic distribution of haplotypes of *T. wilfordii* was less structured and differentiated compared to those of *T. hypoglaucum* (Table 1; Fig. 1a, b). By visual inspection of Fig. 1a, the wild populations distributions of *T. wilfordii* and *T. hypoglaucum* had obviously different characteristics: *T. wilfordii* was mainly restricted to the central range of the two species distributions, whereas *T. hypoglaucum* was across the whole range, being divided into the northeastern group (WJX, WSMX) and the southwestern group (XLH, JSC, GLS, YYW).

Overall, Hap1 is the dominant haplotype of *T. wilfordii*: it was identified in the three wild populations (ETC, FTN, JPX) and the cultivated population (ZXC). Hap2, with only one mutation segregating from Hap1, was identified in an adjacent population (WQM), while a very distinct haplotype (Hap4), with at least 12 mutations segregating from Hap1, was identified in two wild populations (XYY and ZLX) of *T. wilfordii*. All the populations of *T. wilfordii* compose only one haplotype from the specimens we collected. In contrast, the wild populations of *T. hypoglaucum* were much more structured and differentiated: many populations were detected private haplotypes, i.e. the northeastern populations (WJX, WSMX) fixed Hap7 and 8, and the southwestern populations (JSC) fixed Hap3. Interestingly, a wild population of *T. hypoglaucum* (XLH) shared Hap4 with an adjacent wild population of *T. wilfordii* (XYY), which are all located in the central range of the geographic distribution. Due to the geographic relations, we may hypothesize that gene flows occurred more frequently between the two populations from two species.

The cultivated populations of *T. wilfordii* and *T. hypoglaucum* had completely different origins. The cultivated population of *T. wilfordii* (ZXC), located in the northeastern of the distribution, was detected with the same haplotype Hap1 as the wild populations (ETC, FTN, JPX) in the central range, which indicated that cultivated *T. wilfordii* may be originated from at least one of them *ex site* (Table 1; Fig. 1a, b). However, the cultivated population of *T. hypoglaucum* (GJH) was fixed at the haplotypes Hap6 with the local adjacent wild population (GLS) (Table 1; Fig. 1a, b), indicating the cultivated origin *in site* of *T. hypoglaucum.*

**Fig. 2:**
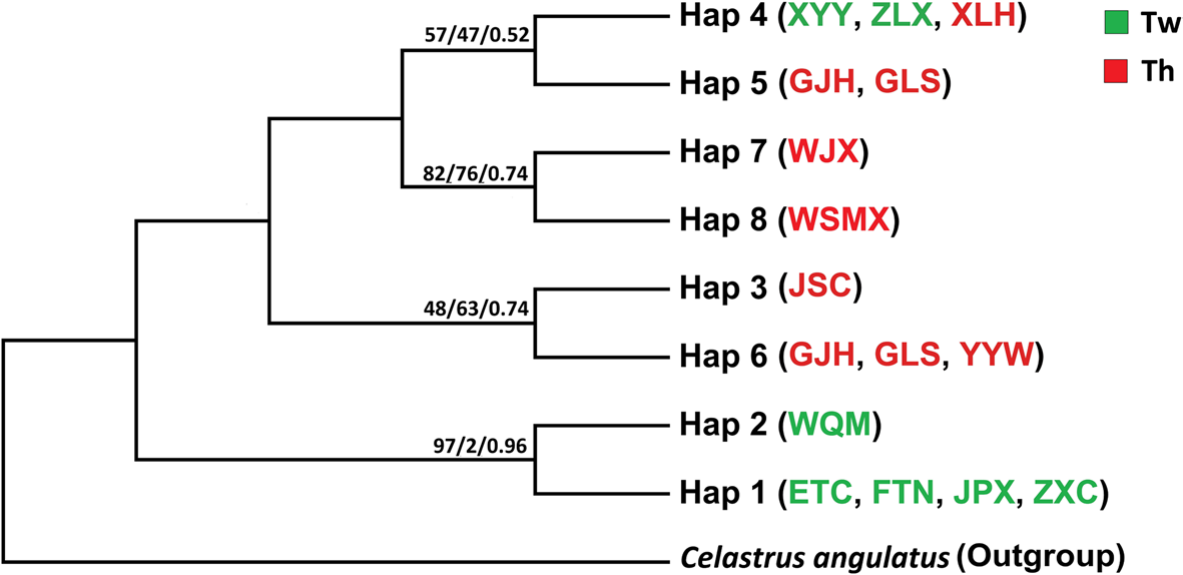
A summarized evolutionary relationship among cpDNA haplotypes of *Tripterygium hypoglaucum *(Th) and *Tripterygium wilfordii* (Tw) inferred from neighbor joining (NJ), maximum parsimony (MP) and Bayesian Inference (BI) methods. The values above the branches indicate bootstrap support values of NJ and MP trees, and Bayesian posterior probabilities (PPs) of BI tree (NJ/MP/BI)

 A total of 14 mutations were identified segregating the two major haplotype clusters: Hap1-2 (dominant in *T. wilfordii*) and Hap5-8 (dominant in *T. hypoglaucum*), which were linked by a shared haplotype (Hap4) (Fig. 1b). The results suggested that the diverging between *T. wilfordii* and *T. hypoglaucum* was significant, while the shared and unresolved haplotypes may need to be further examined by phylogenetic analysis. We, thus, reconstructed gene genealogy using different approaches, i.e. Bayesian Inferences, maximum parsimony and neighbor joining, with cpDNA sequences to elucidate the relations between haplotype clusters (Figure S1). All analyses indicated a consistent phylogeny showing that the eight haplotypes were grouped as two paraphyly lineages (Fig. 2): the basal of the tree was resolved by Hap1and Hap2 while Hap3, 5–8 were further grouped together as descendant lineages. This result further strengthened the previous finding of genetic divergence between the two species (Fig. 1b). The phylogeny also suggested that *T. wilfordii* might have the ancestral state among all the haplotypes, as Hap1 and 2 were exclusive in the species. Hap4, a shared haplotype of the two species, also suggested its transition state during the evolution of the two species based on the genealogy results.

### Genetic diversities and population differentiations were identified using microsatellite loci

In general, microsatellite loci provide more detailed population structures than cpDNA sequences. Therefore, we further analyzed polymorphisms of 10 microsatellite loci to elucidate the population structures of the two species, including 244 individuals. We then estimated 4 different parameters representing population diversities: average numbers of alleles (*Na*), number of effective alleles (*Ne*), observed heterozygosity (*Ho*) and expected heterozygosity (*He*) (Table 3, Table S1 and S2). Among 12 wild and 2 cultivated populations, the average number of alleles (*Na*) is 6.0 for *T. wilfordii* and 12.5 for *T*. *hypoglaucum*. Across all the populations, the number of alleles (*Na*) per locus ranges from 1.4 to 5.0, the number of effective alleles (*Ne*) ranges from 1.40 to 3.52, observed heterozygosity (*Ho*) range from 0.25 to 0.57, and expected heterozygosity (*He*) range from 0.20 to 0.58. Overall, populations of *T. hypoglaucum* showed higher genetic diversities (*Th*: *Na* = 12.5, *Ne* = 4.52) than those of *T. wilfordii* (*Tw*: *Na* = 6.0, *Ne* = 2.32). Nearly all indices indicated that the lowest value was observed in the population of *T. wilfordii*, while the highest value was observed in those of *T. hypoglaucum*, with the exception of the value of *Ho* (both lowest and highest values were found in populations of *T. wilfordii*). However, *T. wilfordii* was found to have higher observed heterozygosity (*Ho* = 0.49) than that of *T. hypoglaucum* (*Ho* = 0.43), which was almost equal to its expected heterozygosity (*He* = 0.51). This indicates that *T. wilfordii* experienced strong gene exchange and significant random mating among populations, but *T. hypoglaucum* had more intense inbreeding and isolation among populations.

**Table 3:** Average number of alleles (*Na*), number of effective alleles (*Ne*), observed heterozygosity (*Ho*) and expected heterozygosity (*He*) of microsatellite loci for two species of *Tripterygium*. ^a^indicates cultivated populations of the two species. Detailed results of all the parameters are shown in Table S1 and S2

Species	Population	*Na*	*Ne*	*Ho*	*He*
*T. wilfordii*	All populations	6.0	2.32	0.49	0.51
	ETC	3.1	1.86	0.45	0.37
	XYY	3.6	2.30	0.37	0.42
	FTN	1.8	1.79	0.74	0.40
	ZXC^a^	1.4	1.40	0.40	0.20
	ZLX	2.3	1.67	0.57	0.33
	JPX	2.0	1.61	0.50	0.30
	WQM	2.5	1.59	0.25	0.32
*T. hypoflaucum*	All populations	12.5	4.52	0.43	0.73
	XLH	3.8	2.21	0.49	0.46
	GJH^a^	3.1	1.82	0.32	0.38
	GLS	3.8	2.26	0.42	0.44
	JSC	2.8	1.80	0.36	0.39
	YYW	5.0	2.57	0.38	0.47
	WJX	3.9	2.72	0.44	0.50
	WSMX	5.0	3.52	0.53	0.58

As expected, the two cultivated populations (ZXC, GJH) significantly reduced genetic diversity compared with the wild populations (Table 3). The lowest numbers of alleles, effective alleles and expected heterozygosity were observed from ZXC (*T. wilfordii*) among all 14 populations. In addition, all the values of genetic diversity (*Na*, *Ne*, *Ho* and *He*) in GJH (*T. hypoglaucum*) were also lower than those of the 6 wild populations of the species.

### Species divergence and population subdivision, particularly in *T. hypoflaucum*, were revealed using both cpDNA and microsatellite loci

As there is no significant difference in morphological characteristics, geographical distribution, and chemical composition between *T. wilfordii* and *T. hypoglaucum*, it is sometimes difficult to distinguish the two species. Here, we combined all the individuals from the two species and performed AMOVA to show the genetic discrepancies between the two species using both chloroplast and nucleus markers. Interestingly, we discovered very different percentages of variations (% variation in Table 4) using different markers.

**Table 4:** Analysis of Molecular Variance (AMOVA) and *F* test of fixation indices based on cpDNA and microsatellite loci for *T. wilfordii* and *T. hypoglaucum*

	**Source of variation**	***d***.***f***.	**Sum of squares**	**Variance components**	**%** **variation**
cpDNA	Among groups	1	269.129	2.59380	48.47
	Among populations within groups	12	413.86	2.74674	51.33
	Within populations	165	1.738	0.01053	0.20
	Total	178	684.726	5.35107	
Microsatellites	Among groups	1	242.112	0.79625	19.48
	Among populations within groups	12	525.638	1.21859	29.81
	Within populations	474	979.896	2.07286	50.71
	Total	487	1747.464	4.08769	

Fixation indices of cpDNA: *F*_*IT*_ = 0.4847; *F*_*ST*_ = 0.9980; *F*_*IS*_= 0.9962

Fixation indices of microsatellites: *F*_*IT*_ = 0.1948; *F*_*ST*_ = 0.4929; *F*_*IS*_ = 0.3702

*F*_*IT*_: overall

*F*_*ST*_: inbreeding coefficient between populations

*F*_*IS*_: inbreeding coefficient within populations

Overall, the variations of cpDNA sequences revealed that only 0.2% of the genetic variations were partitioned within populations, while 51.33% was among populations within each species, and 48.47% was among species (Table 4). This indicates that the populations differ significantly, likely due to genetic divergence of the two species. In addition, we identified very high fixation indices between populations (*F*_*ST*_ = 0.9980) using cpDNA sequences. Unlike cpDNA sequences, polymorphisms in microsatellite loci indicated that 50.71% of the genetic variation was partitioned within populations, 29.81% was among populations within each species, and 19.48% was among species (Table 4). In line with cpDNA sequences, fixation indices estimated by microsatellite loci also suggest the highest inbreeding coefficient between populations in the two species (*F*_*ST*_ =0.4929). Taken together, the results showed the genetic discrepancies among populations, which indicates the differences in the genetics of the two species.

**Fig. 3:**
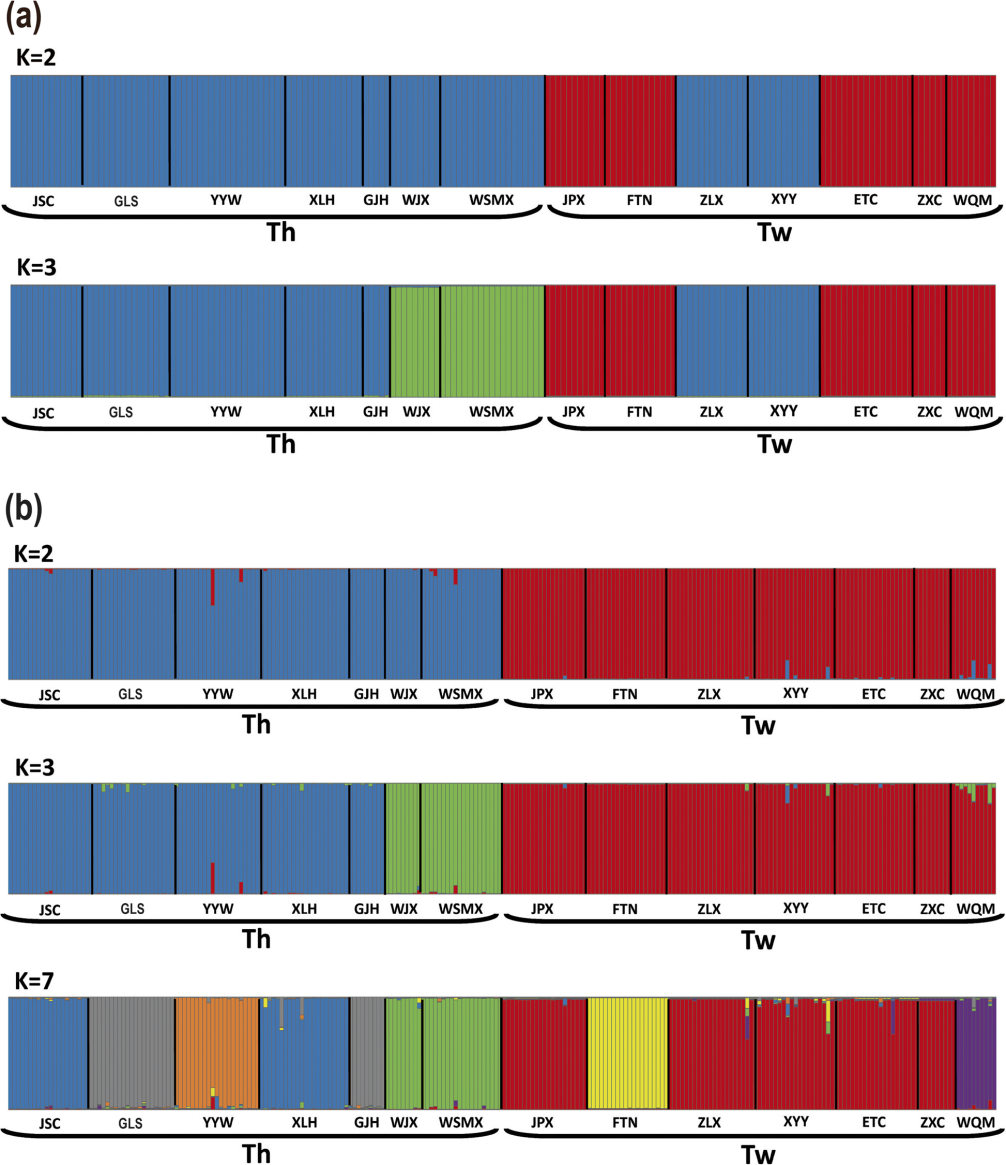
Clustering analysis of (**a**) cpDNA and (**b**) microsatellite loci for *Tripterygium hypoglaucum* (Th) and *Tripterygium wilfordii* (Tw) populations using STRUCTURE. Bold lines distinguish populations, and different colors within each population represent the probability of assigning the individuals to each cluster. The alphabet below the bar indicates the population code

 Genetic structure at a finer resolution, i.e. genetic clustering, was analyzed using STRUCTURE based on both markers (Table S3). Different numbers of clusters (K: estimated number of groups/clusters) were estimated from samples based on the variations in cpDNA sequences or microsatellite loci (Fig. 3). For cpDNA, the best and second best-fit numbers of K values were K = 2 and K = 3 based on ΔK, which indicated that the two species were clustered into two or three groups (ΔK = 993.57 for K = 2 and ΔK = 479.28 for K = 3 based on Bayesian clustering analyses) (Fig. 3a). With an estimation of 2 clusters (K = 2), *T. hypoglaucum* (mostly in blue bars) and *T. wilfordii* (mostly red bars) were divided into two differentiated clusters except for ZLX and XYY populations of *T wilfordii*. The two populations showed closer relations with *T. hypoglaucum*, which is in line with the haplotype analyses: they shared haplotype (Hap4) with XLH of *T. hypoglaucum*. With an estimation of 3 clusters (K = 3), *T. hypoglaucum* was further divided into two clusters corresponding to the geographic subdivisions: populations in the northeast (bars in green) and populations in the southwest (bars in blue).

For microsatellite loci, the optimal estimated number of clusters were 2, 3 and 7, with highest ΔK values 9.28, 5.42 and 9.84, respectively (Table S1). Different from cpDNA, *T. hypoglaucum* and *T. wilfordii* were divided into two distinct groups without exception, in contrast to cpDNA analysis with K = 2 (Fig. 3b). With an estimation of 3 clusters (K = 3), the northeast populations (bars in green) and southwest populations (bars in blue) were able to be distinguished as seen in cpDNA. With an estimated number of 7 clusters showed that more sub-clusters can be identified within each species, i.e. 4 and 3 clusters can be identified in *T. hypoglaucum* and *T. wilfordii*, respectively. Among 4 clusters in *T. hypoglaucum*, the southwest populations (bars in blue when K = 3) were further divided into central-range sub-clusters (bars in blue) and southwest sub-clusters (bars in grey and orange). However, 3 clusters in *T. wilfordii* did not correspond to the geographical structure. This also indicates that a finer scale and resolution of population structures can be identified using microsatellite loci compared to cpDNA haplotypes.

**Fig. 4:**
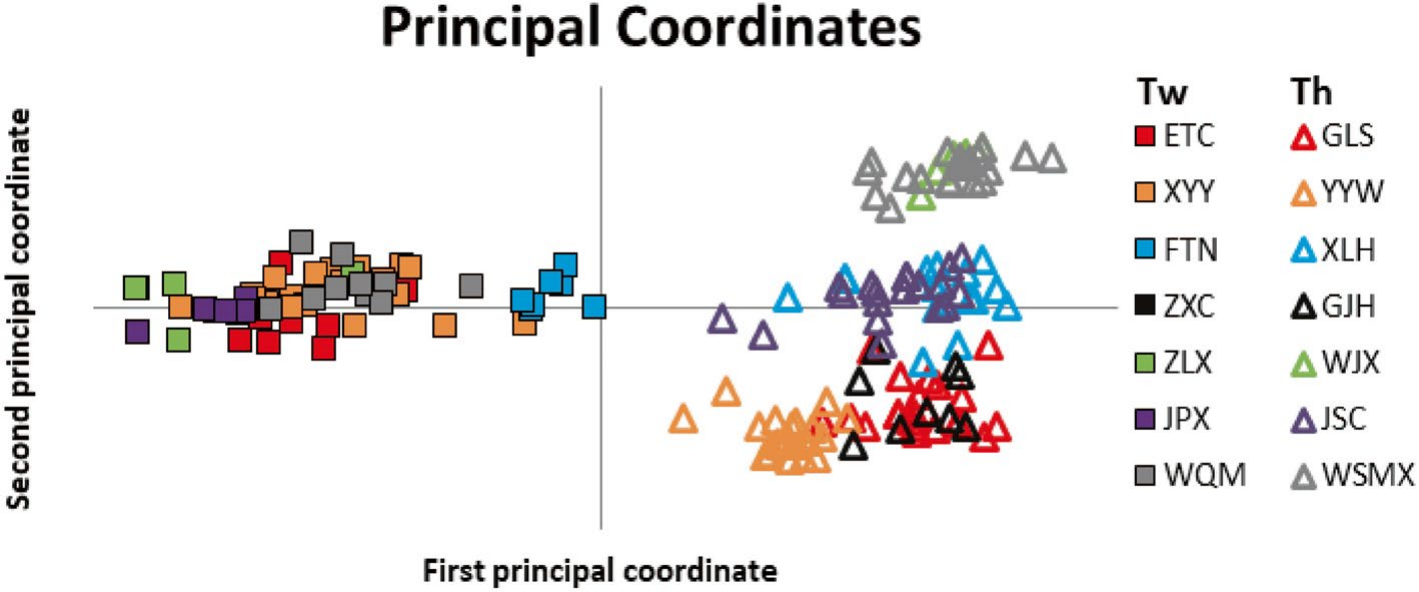
Principal coordinate analysis (PCoA) based on microsatellite data for all populations of *Tripterygium wilfordii *(Tw) and *Tripterygium hypoglaucum *(Th). (The first and second coordinate described 37.92% and 19.33% of total variations, respectively)

 The genetic homogeneity of population samples between *T. wilfordii* and *T. hypoglaucum* was further tested by principal coordinate analysis (PCoA) based on microsatellite data (Fig. 4). At the species level, the genetic compositions of *T. wilfordii* and *T. hypoglaucum* can be obviously distinguished into two clusters at the first axis (explained 37.92% variations) and the second principle (explained 19.33% variations). At the population level, northeast, central-range, and southwest population sub-clusters in *T. hypoglaucum* observed by STRUCTURE analysis were also separated into three groups on the second axis. However, the populations in *T. wilfordii* were not further subdivided into different groups. For cultivated and wild populations of both species, the genetic compositions showed no difference from that of their wild populations at either of the two principles.

Taken together, we demonstrated that different genetic markers and analysis methods all pointed out the genetic divergence of the two species even though they have indistinguishable morphologies and are without geographic isolations.

### Demographic dynamics and historical gene flows between species

As both genetic markers confirmed the species divergence, we would like to ask how the population size fluctuates in the two species and whether there were gene flows between the two species historically.

The marginal distribution of posterior probabilities for demographic parameters was estimated by IMa analyses based on both markers, including the highest posterior parameter estimate (HiPt) and lower to upper bounds of 95% highest posterior densities (HPD) intervals (Table S4, Figure S2). The effective population sizes of *T. hypoglaucum* (*N*_*1*_ = 0.605 and 0.195 from cpDNA and microsatellite markers, respectively) were larger than that of *T. wilfordii* (*N*_*2*_ = 0.195 and 0.054 from cpDNA and microsatellite markers, respectively) (Table S4). However, the effective population sizes of both species are significantly lower than those of their ancestral populations (*N*_*A*_ = 8.461 and 421.445 from cpDNA and microsatellite markers, respectively). This indicates that both species may have undergone population reduction and recently experienced a change in population size again.

The migration estimates between *T. hypoglaucum* and *T. wilfordii* were asymmetric and different using cpDNA and microsatellite loci (Table S4). The cpDNA gene flow from *T. hypoglaucum* to *T. wilfordii* (*M*_*1→2*_ = 0.015, 95% HPD interval: 0.015–5.595) was lesser than that in the opposite direction (*M*_*2→1*_ = 0.075, 95% HPD interval: 0.025–12.025), while gene flow estimated by microsatellite loci showed different results: significantly larger gene flow from *T. hypoglaucum* to *T. wilfordii* (*M*_*1→2*_ = 6.125, 95% HPD interval: 1.625–18.575; *M*_*2→1*_ = 0.813, 95% HPD interval: 1.188–62.813) was detected.

The results showed that shallow genetic exchange could be identified between the two species, which is in line with the results of haplotype diversity: only one haplotype (Hap4) was shared between the two species based on cpDNA sequences. The exchange might be due to closer geographic relations as the two populations (XYY of *T. wilfordii* and XLH of *T. hypoglaucum*) with Hap4 are both in Hunan Province.

## Discussion

The sustainable use of a species relies on properly managing the source plant to avoid over-gathering of the wild population, which may result in severe depletion of the number of individuals and genetic pools [50]. In addition, understanding a species’ genetic compositions and population structures provides the most fundamental knowledge in maintaining their genetic diversities for sustainable use, particularly medicinal plants. In this study, we investigated genetic variations and population differentiations of two widely used medicinal semiwoody vines plants in traditional Chinese medicine, *T. wilfordii* and *T. hypoglaucum*, by using cpDNA and microsatellite loci as genetic markers. From both markers, all the analyses pointed out that the populations of the two species were well grouped into two major clusters according to their species. *T. wilfordii* showed lower genetic diversity of both markers than *T. hypoglaucum*, but it was found to have higher nucleotide divergence of cpDNA and observed heterozygosity of microsatellite loci than those of *T. hypoglaucum.* Further clustering analyses showed that subdivided population structures can be identified in *T. hypoglaucum* across their geographic distribution, i.e. northeast and southwest sub-clusters. In line with our expectation, cultivated populations (ZXC of *T. wilfordii* and GJH of *T. hypoglaucum*) showed a reduction in genetic diversities compared to wild populations, which may result in the loss of the ability to select the strains with high content of medicinal compounds in the long run. This suggests that an urgent effort is required to select individuals from both species to preserve the genetic diversity for future preservation purposes.

The taxonomic investigation suggested one single species in *Tripterygium* because of the indistinguishable morphological differences [30, 35]. DNA barcoding [22] and phytogeographic investigation [34] showed that *T. wilfordii* and *T. hypoglaucum* could be conspecific, but *T. regelii* should be treated separately. However, *T. wilfordii* and *T. hypoglaucum* are two different kinds of materia medica due to different pharmacological activities in traditional Chinese medicine [18, 19, 21], although their identification was difficult by morphological and pharmacognostic methods [18, 37, 51]. In this study, the phylogenetic trees (NJ, MP, and BI) and median-joining network of cpDNA indicated that *T. wilfordii* and *T. hypoglaucum* were clustered into two significant divergent clades except for few shared and unresolved haplotypes (Figs. 1 and 2). Similar results were shown by STRUCTURE analyses based on the cpDNA (Fig. 3). However, significant genetic differentiation between *T. wilfordii* and *T. hypoglaucum* was further revealed by STRUCTURE and PCoA analyses based on microsatellite loci (Figs. 3 and 4). These results suggest that the taxonomic treatments of two species *T. wilfordii* and *T. hypoglaucu* made by Cheng (1999) were justified, which suggested that genetic bases for the content of bioactive compounds of the two species should be different [18, 29]. Phenotypic similarities and shared lesser haplotypes both show a certain degree of similarity between *T. wilfordii and T. hypoglaucum*. However, the more refined distinction of microsatellite marker between *T. wilfordii and T. hypoglaucum* has verified the differentiation of the two species. As for the phenotypic similarities between *T. wilfordii* and *T. hypoglaucum*, the most likely explanation may be the retention of ancestral polymorphisms due to incomplete lineage sorting [52]. Furthermore, the similarities may also be ascribed to pollen-mediated gene flow among the two species and/or phenotypic convergence under similar selection schemes in the same geographical distribution region.

The analysis of migration estimates revealed inconsistent results between *T. hypoglaucum* and *T. wilfordii* when using cpDNA and microsatellites. The results from cpDNA showed that the gene flow intensity from *T. wilfordii* to *T. hypoglaucum* was greater than vice versa. However, microsatellite loci analysis reveals the opposite, indicating a stronger gene flow from *T. hypoglaucum* to *T. wilfordii*. The discrepancies between cpDNA and microsatellite results can be attributed to their different transmission modes and evolutionary rates. Chloroplast DNA, a primarily maternally inherited organelle marker, is dispersed by seeds and evolves at a relatively slow rate, reflecting more historical, long-term gene flow [53, 54]. The phylogenetic trees derived from the study (Fig. 2) further substantiate this inference. In contrast, microsatellite is biparentally inherited, transferred via pollen flow, and evolves faster, reflecting more recent genetic exchanges [53–55]. On the other hand, the difference in effective population sizes can also indirectly provide an explanation for the inconsistency in migration estimates. Evaluations based on effective population sizes reveal that, regardless of cpDNA and microsatellite, the effective population sizes of *T. hypoglaucum* were larger than those of *T. wilfordii*. This characteristic could enable greater gene flow for *T. hypoglaucum*, thus explaining the microsatellite analysis results. Additionally, the cultivation method of *T. wilfordii* is mainly wild transplantation, seedling cultivation, and branch-cutting seedling transplantation. Unlike *T. wilfordii*, *T. hypoglaucum* is mainly propagated by cutting seedlings. These distinct reproduction methods may also lead to different gene flow patterns.

From the literature, the mean value of cpDNA haplotype diversity (*Hd*) detected by various cpDNA markers is 0.67 in 170 species of plants compiled by Petit et al. (2005) [53]. The cpDNA haplotype diversity in *T. wilfordii* (*Hd =* 0.566) was low relative to those plants and was significantly lower than that in *T. hypoglaucum* (*Hd =* 0.828). *Tripterygium wilfordii and T. hypoglaucum* are plants in an archaic taxon, Celastraceae, with a long evolutionary history. Unlike other species in Celastraceae, e.g. *Dipentodon sinicus*, showing very high haplotype diversity (*Hd* = 0.902) [56], *T. wilfordii* showed unexpectedly low haplotype diversity in this study. The possible reason might be the over-exploitation due to the heavy usage of the species for medicinal purposes. Demographic dynamics also showed that *T. wilfordii* has undergone the recent contraction of effective population sizes (Table S5, Fig. 2). Therefore, there is an urgent need to develop conservation and cultivation practices for *T. wilfordii*.

Cultivation of wild plants always produces genetic bottlenecks and thus results in loss of genetic diversity due to founder effects and unconscious or conscious selections [7]. The cultivation of *Tripterygium* plants originated from local farmers’ practice in traditional agricultural habitats with possibly less than fifty years of history [57]. Compared with long-term and large-scale cultivated medicinal plants in China (e.g., *Coptis chinensis* var. *chinensis*; *Scrophularia ningpoensis*) [58, 59], recently cultivated medicinal plants used to have larger population size and thus likely be able to maintain greater genetic diversity due to multiple origins and shorter artificial selection time (e.g., *Scutellaria baicalensis*; *Magnolia officinalis* var. *biloba*) [60, 61]. However, in the study, significantly lower genetic diversity of cultivated *T. wilfordii* and *T. hypoglaucum* was revealed: the main values of genetic diversity of microsatellite loci (*Na*, *Ne* and *He*) in cultivated *T. wilfordii* were the lowest among all the 14 populations, and these values of cultivated *T. hypoglaucum* were also lower than those of its 6 wild populations (Table 3). These results may be explained by the small initial population size and vegetative propagation during the cultivation of the two species. First, The geographical distribution of cpDNA haplotypes indicates that only one haplotype (Hap1) and two haplotypes (Hap5 and 6) were brought into cultivated populations of *T. wilfordii* and *T. hypoglaucum*, respectively, from at least one wild population. (Fig. 1; Table 1). The evidence supports that cultivated *T. wilfordii* or *T. hypoglaucum* could have originated from one place for a single time, resulting in a small initial population size during cultivation. Second, cultivation of *Tripterygium* plants was mainly from cuttage because of several issues such as the cost of seed gathering, lower germination rate, and investment in factory equipment [57]. This cultivation practice inevitably led to a shift from sexually reproducing wild populations to vegetatively propagating cultivated populations [62, 63] and a significant reduction of genetic diversity in the cultivated populations [12, 63].

The decline of genetic diversity in cultivation not only results in a narrow genetic range of material to meet breeding objectives such as selections for high-yielding or pharmacological standard individuals but also might cause the loss of evolutionary potential of cultivated species due to inbreeding. These experiences were revealed by many crop species [64, 65] and a few medicinal plants, such as *Scrophularia ningpoensis* [58]. Therefore, the integrity of genetic diversity is urgently needed to be conservated during the cultivation of *Tripterygium* plants.

Facing the rapidly growing demands for materia medica of *T. wilfordii* for its new pharmacological activities, the sustainable utilization of *T. wilfordii* resources has become an urgent problem that needs to be solved. The study on the genetic diversity pattern of *T. wilfordii* and its sibling species *T. hypoglaucum* is the precondition for the sustainable utilization of *T. wilfordii* resources. Our results suggest that the taxonomic treatments of two species *T. wilfordii* and *T. hypoglaucum* made by Cheng (1999) are justified [29], which provides genetic bases for the contents of bioactive compounds of the two species being different. Over-exploitation might result in significantly lower genetic diversity and less subdivided genetic structure of *T. wilfordii* than those of *T. hypoglaucum.* During the course of cultivation of the two species, genetic diversity should be increased and the introduction and cultivation *ex site* should be avoided. This study not only provides baseline data for protecting and utilizing the genetic resource of *T. wilfordii* through a conservation-by-cultivation approach but also represents a paradigm for instructing future cultivation projects of important medicinal plants with new pharmacological activities.

## Materials and methods

### Sampling, DNA extraction and microsatellite genotyping

Field investigations were conducted throughout the distribution range of *T. wilfordii* and *T. hypoglaucum*. A total of 244 individuals representing 7 *T. wilfordii* and 7 *T. hypoglaucum* populations were collected (Table 1; Fig. 1a). Five to twenty-two individuals were sampled for each population, and the distances of each population were more than 50 km. Fresh leaves of each individual were collected and dried with silica gel for DNA extraction. Dr. Lisong Wang conducted the formal identification of the voucher specimens, and voucher specimens have been deposited in the herbaria of the Institute of Chinese Materia Medica (CMMI), China Academy of Chinese Medical Sciences. The precise geographic location of each sampled population was determined using a Garmin GPS unit. The distribution map was generated using Esri’s ArcGIS v9.3 software, incorporating the ESRI World Elevation GMTED2010 dataset and source from USGS.

Total genomic DNA was extracted using a modified cetyltrimethyl ammonium bromide (CTAB) protocol [66]. The relative purity and concentration of extracted DNA were estimated by ethidium bromide staining on agarose gels compared with known DNA concentration markers. Two cpDNA regions *psb*A-*trn*H and *trn*L-*trn*F were chosen for amplifcation and sequencing using the primers indicated in Taberlet et al. (1991) and Sang et al. (1997) [67, 68], respectively (Table S5). PCR amplifications were carried out in a volume of 20 µL using 1 µL of template DNA (50–100 ng), 2 µL of 10×reaction buffer, 1.6 µL dNTP mix (2.5 mM), 1.25 µL 10 µM of each primer, 0.2 µL Ex-Taq DNA polymerase (Takara Shuzo Co., Ltd., Otsu, Japan), 12.7 µL sterile distilled water. Reactions run on a Veriti thermocycler (Applied Biosystems, USA). The protocol for *psb*A-*trn*H consisted of one cycle of denaturation at 95 ºC for 5 min; 35 cycles of 1 min denaturation at 94 ºC, 1 min of annealing at 55 ºC and 1 min 30 s of extension at 72 ºC; followed by an 8 min extension at 72ºC. The protocol for *trn*L-*trn*F consisted of one cycle of denaturation at 94 ºC for 4 min; 35 cycles of 45 s denaturation at 94 ºC, 30 s annealing at 58 ºC, and 1 min 30 s extension at 72 ºC; then 7 min extension at 72 ºC. All the purified PCR products were sequenced directly in both directions on an ABI 3730XL automated sequencer (Applied Biosystem, Foster City, CA).

A total of 10 microsatellite primer pairs derived from Novy & Jones (2011) [69] were chosen for this study (Table S6). Amplification of DNA was carried out in 25 µL reactions consisting of 1 µL DNA, 2.5 µL of 10×reaction buffer, 2 µL dNTP (2.5mM), 0.4 µL each primer, and 0.25 µL rTaq DNA polymerase (5U/µL), 18.45 µL sterile distilled water. The reaction was optimized and programmed on a PCR System 9700 (Applied Biosystems GeneAmp, USA) for one cycle of denaturation at 94 ºC for 5 min, 40 cycles of 30 s denaturation at 94 ºC, 30 s at proper annealing temperatures, and 30 s extension at 72 ºC; then 10 min extension at 72 ºC. The PCR amplified products were scored using an ABI 3730XL automated sequencer using a 50-cm capillary, polymerPOP-7 and ROX 500 (both Applied Biosystems) as an internal standard. Fragment sizes were assessed using GeneMarker software version 1.5. Allele size is indicated twice manually to reduce scoring error.

### Sequence alignment, population genetics statistics, and genealogy of cpDNA

All 244 samples were used in analyses of microsatellite markers (SSRs), and only 179 were used in cpDNA sequencing analyses because partial samples could not be sequenced due to degradation (Table 1). All sequences were edited and assembled by SeqMan 7.1.0 [70] and BioEdit ver. 7.0.9 [71] with manual correction. Sequence alignments were performed with CLUSTALX version 2.0 [72] and then manually modified. Two cpDNA regions were combined and treated as a single marker. Summary statistics, including the number of haplotypes (*Nh*) and haplotype diversity (*Hd*) were determined. The level of genetic diversity within populations was calculated by measures of nucleotide divergence, *θ*_*W*_ [73] and *π* [74, 75], using DnaSP Version 5.10.1 [76]. The signatures of demographic change were examined by neutrality tests using DnaSP, including Tajima’s *D* statistic [77], Fu and Li’s D* statistic [78], Fu and Li’s F* tests. An analysis of molecular variance (AMOVA) [79] was conducted to estimate the genetic variation within and among populations grouped by species by using the program Arlequin v.3.5.1.2 [80].

Phylogenetic relationships among cpDNA haplotypes of *T. wilfordii* and *T. hypoglaucum* were inferred using maximum parsimony (MP) in PAUP* 4.0b10 [81] and Bayesian Inference (BI) implemented in MrBayes v. 3.1.2 [82, 83] with *Celastrus angulatus* (Celastraceae) as the outgroup. For BI inference, the normal prior probability distribution was chosen as prior. All parameters once every 1000 steps from 10^7^ Markov coupled Markov chain (MCMC) steps, with the first 10% of samples discarded as burn-in. The TRACER program v.1.6 [84] was used to examine the convergence of chains to the stationary distribution. Trees were then compiled as maximum clade credibility trees by TREEANNOTATOR v.1.7.5 [85] and visualized by FIGTREE v.1.4 [86] to display the highest posterior density (HPD) intervals at 95% (upper and lower) for each node. Neighbor joining (NJ) phylogeny relationships of cpDNA haplotypes that was based on the neighbor-joining method were constructed using MEGA v. 7.02 [26]. The degree of relatedness among cpDNA haplotypes was also estimated using Network v. 4.2.0.1 [87]. with indels that were treated as single mutational events.

### Population genetic analysis and gene flow

For each microsatellite locus, genetic diversity was assessed by the average number of alleles (*Na*), the number of effective alleles (*Ne*), the observed heterozygosity (*Ho*) and the expected heterozygosity (*He*), which were estimated by using GenAlEx v.6.5 [88]. The population structure was evaluated to estimate the distinction between the two species by calculating the population differentiation level (Weir and Cockerham’s *F*_*ST*_) [89] in AMOVA analysis implemented in Arlequin v.3.5.1.2., partitioning the genetic diversity into three levels: among species (groups), among populations within species (groups) and within populations. Significance tests were conducted using 10,000 permutations. Pairwise *F*_*ST*_ among populations and species were also estimated hierarchically in the program DnaSP. The principal coordinate analysis (PCoA) was also conducted using GenAlEx 6.5. Genetic groups were inferred using a Bayesian clustering method implemented in STRUCTURE v.2.3.4 [90]. by using both cpDNA and microsatellite loci. A total of 20 independent simulations were run for each (K = 1–15) with 500 000 burn-in steps followed by 500 000 MCMC steps using the admixture model with correlated allelic frequencies [91] because hybridization events are common in plants [92]. The true number of K was determined using the method of Pritchard et al. (2000) [90] and Evanno et al. (2005) [93]. Structure Harvester v.0.6.8 [94] was used to visualize the STRUCTURE output.

The rates of gene flow between *T. wilfordii* and *T. hypoglaucum* (*M*) and effective population size (*N*) based on cpDNA sequences and microsatellite loci were estimated by using IMa2 software [95]. The IMa analyses were performed using > 3 million burn-in steps per run to ensure all parameters were well mixed, and additional genealogies were saved until all effective sample sizes of parameters were > 100. Three independent runs with random seeds were performed to check the consistency between results. The final results were calculated by averaging the values estimated in the three runs. A swapping procedure was also used to enhance the mixing of chains using a geometric heating scheme with 15 parallel chains. All parameters were scaled by the mutation rate of 1.52 × 10^−9^ (1.0 × 10^−9^ − 3.0 × 10^−9^) substitutions per year for cpDNA by Wolfe et al. (1987) and Richardson & Stojiljkovic (2001) and 4.76 × 10^−3^ (1.2 × 10^−4^ − 2.7 × 10^−2^) per allele per generation for microsatellite loci by Cieslarová et al. (2011) [42, 96, 97].

### Supplementary Information

**Supplementary Material 1.**

**Supplementary Material 2.**

## Data Availability

All sequences for this study have been uploaded to the NCBI GenBank as follows: Sequences of cpDNA (*psb*A-*trn*H and *trn*L-*trn*F) were obtained from a total of 179 individuals and submitted to GenBank with the following accession numbers: PP128529-PP128707 (https://www.ncbi.nlm.nih.gov/nuccore/PP128529 to. https://www.ncbi.nlm.nih.gov/nuccore/PP128707) for *psb*A-*trn*H and PP128708-PP128886 for *trn*L-*trn*F. (https://www.ncbi.nlm.nih.gov/nuccore/PP128708 to. https://www.ncbi.nlm.nih.gov/nuccore/PP128886)
