# Supplementary material for: Genetic diversities in wild and cultivated populations of the two closely-related medical plants species, Tripterygium Wilfordii and T. Hypoglaucum (Celastraceae)
Source: BMC Plant Biol. 2024 Mar 16;24:195. doi: 10.1186/s12870-024-04826-x (PMC10944624; doi:10.1186/s12870-024-04826-x)
Supplement: Supplementary file 1 — Supplementary Material 1. [file 12870_2024_4826_MOESM1_ESM.docx]

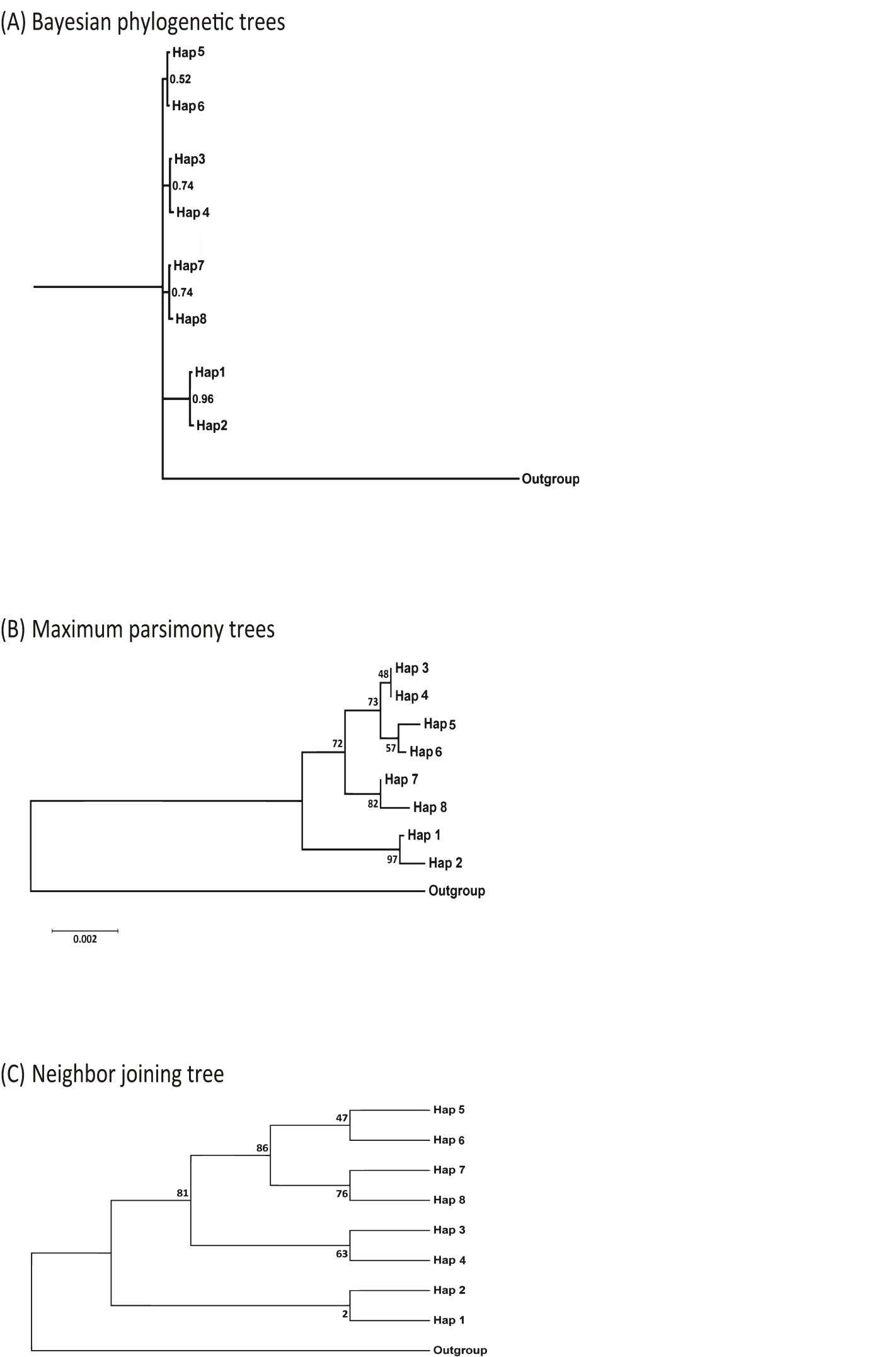


**Fig. S1** Gene genealogy of haplotypes constructed by three different approaches: Bayesian Inferences (**a**), Maximum parsimony (**b**), and Neighbor joining (**c**).

**Fig. S2** Marginal distribution of posterior probabilities for demographic parameters estimated by IMa analyses based on cpDNA sequences (**a-c**) and microsatellite loci (**d-f**). The posterior distributions for two current effective population sizes (*N_1_* and *N_2_*), ancestral effective population size (*N_A_*), and migration rate (*M_1_*→*M_2_* and *M_1_*←*M_2_*) between *T. hypoglaucum* (*M_1_*) and *T. wilfordii* (*M_2_*) were shown. All parameters were scaled by a mutation rate of 1.52 × 10^−9^ substitutions per year for chloroplast noncoding regions by Wolfe *et al*. (1987) and a mutation rate of 4.76 × 10^-3^ 3 per locus per generation for microsatellite loci by Cieslarová *et al*. (2011).
