# Supplementary material for: Genetic diversities in wild and cultivated populations of the two closely-related medical plants species, Tripterygium Wilfordii and T. Hypoglaucum (Celastraceae)
Source: BMC Plant Biol. 2024 Mar 16;24:195. doi: 10.1186/s12870-024-04826-x (PMC10944624; doi:10.1186/s12870-024-04826-x)
Supplement: Supplementary file 2 — Supplementary Material 2. [file 12870_2024_4826_MOESM2_ESM.docx]

**Table S1** Estimates of genetic diversity by 10 polymorphic microsatellite loci for *Tripterygium wilfordii*, number of different alleles (*Na*), number of effective alleles (*Ne*), observed heterozygosity (*Ho*) and expected heterozygosity (*He*) are provided.

|  |  | JSC | | | |  | GLS | | | |  | YYW | | | |  | XLH | | | |
| --- | --- | --- | --- | --- | --- | --- | --- | --- | --- | --- | --- | --- | --- | --- | --- | --- | --- | --- | --- | --- |
| Locus |  | *Na* | *Ne* | *Ho* | *He* |  | *Na* | *Ne* | *Ho* | *He* |  | *Na* | *Ne* | *Ho* | *He* |  | *Na* | *Ne* | *Ho* | *He* |
| P1 |  | 2 | 1.84 | 0.5 | 0.46 |  | 6 | 3.64 | 0.67 | 0.73** |  | 8 | 3.32 | 0.8 | 0.70*** |  | 5 | 2.96 | 0.77 | 0.66** |
| P2 |  | 2 | 1.47 | 0.4 | 0.32 |  | 7 | 4.34 | 0.9 | 0.77** |  | 6 | 2.85 | 0.67 | 0.65*** |  | 5 | 3.02 | 0.73 | 0.67** |
| P3 |  | 4 | 2.69 | 0.4 | 0.63 |  | 6 | 3.02 | 0.67 | 0.67** |  | 5 | 1.98 | 0.24 | 0.50*** |  | 3 | 1.26 | 0.18 | 0.21** |
| P4 |  | 4 | 1.6 | 0.45 | 0.37 |  | 3 | 1.4 | 0.33 | 0.28** |  | 12 | 7.29 | 0.67 | 0.86*** |  | 7 | 3.23 | 0.64 | 0.69** |
| P5 |  | 3 | 2.8 | 0.55 | 0.64 |  | 1 | 1 | - | - |  | 4 | 1.42 | 0.33 | 0.29*** |  | 4 | 2.49 | 0.77 | 0.6*** |
| P6 |  | 3 | 1.5 | 0.3 | 0.34 |  | 1 | 1 | - | - |  | 1 | 1 | - | - |  | 5 | 2.32 | 0.45 | 0.57** |
| P7 |  | 1 | 1 | - | - |  | 4 | 1.65 | 0.29 | 0.40** |  | 4 | 2.22 | - | 0.55*** |  | 3 | 2.6 | 0.55 | 0.62** |
| P8 |  | 3 | 1.23 | 0.2 | 0.18 |  | 3 | 2.71 | 0.62 | 0.63** |  | 2 | 1.05 | 0.05 | 0.05*** |  | 4 | 2.27 | 0.8 | 0.56** |
| P9 |  | 3 | 2.11 | 0.5 | 0.53 |  | 2 | 1.45 | 0.19 | 0.31** |  | 2 | 1.8 | 0.48 | 0.44*** |  | 1 | 1 | - | - |
| P10 |  | 3 | 1.73 | 0.3 | 0.42 |  | 5 | 2.34 | 0.57 | 0.57** |  | 6 | 2.77 | 0.52 | 0.64*** |  | 1 | 1 | - | - |

Note : Chi-Square Tests for Hardy-Weinberg Equilibrium: * P<0.05, ** P<0.01, *** P<0.001

**Table S1 (Continue)** Estimates of genetic diversity by 10 polymorphic microsatellite loci for *Tripterygium wilfordii*, number of different alleles (*Na*), number of effective alleles (*Ne*), observed heterozygosity (*Ho*) and expected heterozygosity (*He*) are provided.

|  |  | GJH | | | |  | WJX | | | |  | WSMX | | | |
| --- | --- | --- | --- | --- | --- | --- | --- | --- | --- | --- | --- | --- | --- | --- | --- |
| Locus |  | *Na* | *Ne* | *Ho* | *He* |  | *Na* | *Ne* | *Ho* | *He* |  | *Na* | *Ne* | *Ne* | *He* |
| P1 |  | 6 | 2.79 | 0.56 | 0.64* |  | 10 | 7.36 | 0.67 | 0.86** |  | 16 | 11.59 | 11.59 | 0.91*** |
| P2 |  | 3 | 1.78 | 0.33 | 0.44* |  | 3 | 1.98 | 0.67 | 0.49** |  | 4 | 3.86 | 3.86 | 0.74*** |
| P3 |  | 5 | 2.1 | 0.33 | 0.52* |  | 4 | 4 | 0.38 | 0.75** |  | 6 | 4.06 | 4.06 | 0.75*** |
| P4 |  | 3 | 1.26 | 0.22 | 0.2** |  | 4 | 2.05 | 0.22 | 0.51** |  | 4 | 1.76 | 1.76 | 0.43*** |
| P5 |  | 1 | 1 | - | - |  | 3 | 1.41 | 0.33 | 0.29** |  | 5 | 3.09 | 3.09 | 0.68*** |
| P6 |  | 1 | 1 | - | - |  | 5 | 2.89 | 0.56 | 0.65** |  | 3 | 2.9 | 2.9 | 0.66*** |
| P7 |  | 3 | 1.78 | 0.33 | 0.44* |  | 2 | 1.25 | - | 0.20** |  | 3 | 2.51 | 2.51 | 0.6**** |
| P8 |  | 3 | 2.79 | 0.67 | 0.64* |  | 3 | 2.22 | 1 | 0.55** |  | 2 | 1.1 | 1.1 | 0.1**** |
| P9 |  | 3 | 2.16 | 0.33 | 0.54* |  | 4 | 3 | 0.56 | 0.67** |  | 4 | 3.01 | 3.01 | 0.67*** |
| P10 |  | 3 | 1.57 | 0.44 | 0.36* |  | 1 | 1 | - | - |  | 3 | 1.29 | 1.29 | 0.22*** |

Note : Chi-Square Tests for Hardy-Weinberg Equilibrium: * P<0.05, ** P<0.01, *** P<0.001

**Table S2** Estimates of genetic diversity by 10 polymorphic microsatellite loci for *Tripterygium hypoglaucum*, number of different alleles (*Na*), number of effective alleles (*Ne*), observed heterozygosity (*Ho*) and expected heterozygosity (*He*) are provided.

|  |  | JPX | | | |  | FTN | | | |  | ZLX | | | |  | XYY | | | |
| --- | --- | --- | --- | --- | --- | --- | --- | --- | --- | --- | --- | --- | --- | --- | --- | --- | --- | --- | --- | --- |
| Locus |  | *Na* | *Ne* | *Ho* | *He* |  | *Na* | *Ne* | *Ho* | *He* |  | *Na* | *Ne* | *Ho* | *He* |  | *Na* | *Ne* | *Ho* | *He* |
| P1 |  | 4 | 2.23 | 0.71 | 0.55*** |  | 2 | 1.98 | 0.8 | 0.50*** |  | 3 | 2.19 | 0.95 | 0.54*** |  | 8 | 4.47 | 0.8 | 0.78*** |
| P2 |  | 1 | 1 | - | - |  | 2 | 2 | 0.95 | 0.50*** |  | 2 | 2 | 0.91 | 0.50*** |  | 3 | 1.58 | 0.25 | 0.37*** |
| P3 |  | 1 | 1 | - | - |  | 2 | 2 | 0.95 | 0.50*** |  | 1 | 1 | - | - |  | 2 | 1.1 | - | 0.10*** |
| P4 |  | 2 | 1.89 | 0.76 | 0.47** |  | 1 | 1 | - | - |  | 4 | 2.29 | 1 | 0.56*** |  | 6 | 4.88 | 0.9 | 0.8*** |
| P5 |  | 1 | 1 | - | - |  | 2 | 2 | 1 | 0.50*** |  | 2 | 1.05 | 0.05 | 0.04*** |  | 3 | 1.46 | 0.26 | 0.31*** |
| P6 |  | 4 | 2.07 | 0.76 | 0.52*** |  | 2 | 1.95 | 0.83 | 0.49*** |  | 2 | 1.96 | 0.86 | 0.49*** |  | 3 | 1.49 | 0.2 | 0.33*** |
| P7 |  | 1 | 1 | - | - |  | 2 | 2 | 0.95 | 0.50*** |  | 1 | 1 | - | - |  | 2 | 1.22 | 0.1 | 0.18*** |
| P8 |  | 2 | 2 | 1 | 0.50*** |  | 2 | 2 | 0.95 | 0.50*** |  | 2 | 1.1 | 0.09 | 0.09 |  | 1 | 1 | - | - |
| P9 |  | 2 | 1.89 | 0.76 | 0.47*** |  | 2 | 2 | 0.95 | 0.50*** |  | 2 | 1.98 | 0.91 | 0.50*** |  | 4 | 3.11 | 0.65 | 0.68*** |
| P10 |  | 2 | 2 | 0.95 | 0.50*** |  | 1 | 1 | - | - |  | 4 | 2.17 | 0.91 | 0.54*** |  | 4 | 2.64 | 0.5 | 0.62*** |

Note : Chi-Square Tests for Hardy-Weinberg Equilibrium: * P<0.05, ** P<0.01, *** P<0.001

**Table S2 (Continue)** Estimates of genetic diversity by 10 polymorphic microsatellite loci for *Tripterygium hypoglaucum*, number of different alleles (*Na*), number of effective alleles (*Ne*), observed heterozygosity (*Ho*) and expected heterozygosity (*He*) are provided.

|  |  | ETC | | | |  | ZXC | | | |  | WQM | | | |
| --- | --- | --- | --- | --- | --- | --- | --- | --- | --- | --- | --- | --- | --- | --- | --- |
| Locus |  | *Na* | *Ne* | *Ho* | *He* |  | *Na* | *Ne* | *Ho* | *He* |  | *Na* | *Ne* | *Ho* | *He* |
| P1 |  | 5 | 3.11 | 0.95 | 0.68*** |  | 5 | 2 | 1 | 0.50** |  | 2 | 1.37 | 0.2 | 0.27** |
| P2 |  | 2 | 1.34 | 0.2 | 0.26*** |  | 2 | 1 | - | - |  | 1 | 2.11 | 0.6 | 0.53** |
| P3 |  | 1 | 1 | - | - |  | 1 | 1 | - | - |  | 1 | 1 | - | - |
| P4 |  | 6 | 1.45 | 0.25 | 0.31*** |  | 6 | 2 | 1 | 0.50** |  | 2 | 1.83 | 0.3 | 0.46** |
| P5 |  | 2 | 1.1 | - | 0.10*** |  | 2 | 1 | - | - |  | 1 | 1.72 | - | 0.42** |
| P6 |  | 3 | 1.23 | 0.05 | 0.18*** |  | 3 | 1 | - | - |  | 1 | 1.1 | 0.1 | 0.1*** |
| P7 |  | 2 | 1.72 | 0.6 | 0.42*** |  | 2 | 1 | - | - |  | 1 | 1.47 | 0.4 | 0.32** |
| P8 |  | 2 | 1.78 | 0.65 | 0.44*** |  | 2 | 1 | - | - |  | 1 | 2.2 | 0.5 | 0.55** |
| P9 |  | 3 | 2.78 | 0.95 | 0.64*** |  | 3 | 2 | 1 | 0.50** |  | 2 | 1.94 | 0.3 | 0.49** |
| P10 |  | 5 | 3.05 | 0.8 | 0.67*** |  | 5 | 2 | 1 | 0.50** |  | 2 | 1.1 | 0.1 | 0.1*** |

Note : Chi-Square Tests for Hardy-Weinberg Equilibrium: * P<0.05, ** P<0.01, *** P<0.001

**Table S3** Mean LnP (K) and ΔK for clusters using Bayesian assignment test in STRUCTURE to infer the number of clusters for all populations of *Tripterygium wilfordii* and *Tripterygium hypoglaucum*.

|  | cpDNA | |  | Microsatellite loci | |
| --- | --- | --- | --- | --- | --- |
| K | Mean LnP (K) | ΔK |  | Mean LnP (K) | ΔK |
| 1 | -1060.81 | **-** |  | -8439.46 | **-** |
| 2 | -389.05 | **993.57** |  | -6821.48 | **9.28** |
| 3 | -180.48 | **479.28** |  | -6035.32 | **5.42** |
| 4 | -131.71 | 2.47 |  | -5568.36 | 0.90 |
| 5 | -147.03 | 10.28 |  | -5237.08 | 0.45 |
| 6 | -172.11 | 5.58 |  | -4997.4 | 0.19 |
| 7 | -179.76 | 3.57 |  | -4704.33 | **9.84** |
| 8 | -208.99 | 2.39 |  | -4814.18 | 0.36 |
| 9 | -268.05 | 5.64 |  | -4473.6 | 0.68 |
| 10 | -257.03 | 1.82 |  | -4654.31 | 0.26 |
| 11 | -260.86 | 0.44 |  | -4345.84 | 2.30 |
| 12 | -272.24 | 1.24 |  | -4707.33 | 0.84 |
| 13 | -297.52 | 0.06 |  | -4465.52 | 1.48 |
| 14 | -321.44 | 0.48 |  | -4745.27 | 0.26 |
| 15 | -334.93 | **-** |  | -4725.34 | **-** |

Note: Values in bold type indicate the high proportion of membership for each population that we used in this study.

**Table S4** The highest posterior parameter estimate (HiPt) and lower to upper bounds of 95% HPD intervals of demographic parameters of *T. hypoglaucum* (*M_1_*) and *T. wilfordii* (*M_2_*) estimated by IMa analysis.

|  | |  | *N_1_* | *N_2_* | *N_A_* | *M_1→2_* | *M**_2→1_* |
| --- | --- | --- | --- | --- | --- | --- | --- |
| cpDNA | Raw data | HiPt | 0.605 | 0.195 | 8.461 | 0.015 | 0.075 |
|  |  | 95% HPDLo | 0.215 | 0.039 | 4.556 | 0.015 | 0.025 |
|  |  | 95% HPDHi | 2.636 | 1.601 | 1012.053 | 5.595 | 12.025 |
|  | $\mu=1\times{10}^{-9}$ | HiPt | 7,565,988 | 2,440,641 | 1.06E+08 | 0.000 | 0.000 |
|  |  | 95% HPDLo | 2,684,705 | 488,128.2 | 56,948,294 | 0.000 | 0.000 |
|  |  | 95% HPDHi | 32,948,656 | 20,013,258 | 1.27E+10 | 0.000 | 0.000 |
|  | $\mu=1.52\times{10}^{-9}$ | HiPt | 4,977,623 | 1,605,685 | 69,579,683 | 0.000 | 0.000 |
|  |  | 95% HPDLo | 1,766,253 | 321,137 | 37,465,983 | 0.000 | 0.000 |
|  |  | 95% HPDHi | 21,676,747 | 13,166,617 | 8.32E+09 | 0.000 | 0.000 |
|  | $\mu=3\times{10}^{-9}$ | HiPt | 2,521,996 | 813,547.1 | 35,253,706 | 0.000 | 0.000 |
|  |  | 95% HPDLo | 894,901.8 | 162,709.4 | 18,982,765 | 0.000 | 0.000 |
|  |  | 95% HPDHi | 10,982,885 | 6,671,086 | 4.22E+09 | 0.000 | 0.001 |
| microsatellite | Raw data | HiPt | 0.195 | 0.054 | 421.445 | 6.125 | 0.813 |
|  |  | 95% HPDLo | 0.117 | 0.018 | 323.018 | 1.625 | 1.188 |
|  |  | 95% HPDHi | 0.899 | 0.519 | 602.192 | 18.575 | 62.813 |
|  | $\mu=1.2\times{10}^{-4}$ | HiPt | 10.262 | 2.820 | 22,134.722 | 0.030 | 0.004 |
|  |  | 95% HPDLo | 6.155 | 0.940 | 16,965.252 | 0.008 | 0.006 |
|  |  | 95% HPDHi | 47.206 | 27.258 | 31,627.747 | 0.088 | 0.300 |
|  | $\mu=4.76\times{10}^{-3}$ | HiPt | 407.083 | 111.875 | 878,010.625 | 0.001 | 0.000 |
|  |  | 95% HPDLo | 244.167 | 37.291 | 672,955 | 0.001 | 0.000 |
|  |  | 95% HPDHi | 1,872.5 | 1,081.25 | 1,254,567.292 | 0.002 | 0.008 |
|  | $\mu=2.7\times{10}^{-2}$ | HiPt | 1.809 | 0.497 | 3,902.269 | 0.165 | 0.022 |
|  |  | 95% HPDLo | 1.085 | 0.166 | 2,990.911 | 0.044 | 0.032 |
|  |  | 95% HPDHi | 8.322 | 4.805 | 5,575.854 | 0.501 | 1.696 |

Note: *N_1_* is the effective population size of *Tripterygium hypoglaucum*, N_2_ is the effective population size of *Tripterygium wilfordii*, *N_A_* is the effective population size of the ancestral, *M_1→2_* is the migration rate from *Tripterygium hypoglaucum* to *Tripterygium wilfordii*, *M_2→1_* is the migration rate from *Tripterygium wilfordii* to *Tripterygium hypoglaucum.* The parameter are scaled by the mutation rate of Richardson *et al.* (2001) and Wolfe *et al.* (1987) for cpDNA and Cieslarová *et al.* (2011) for microsatellite. The parameter are scaled by the mutation rate of 1.52 × 10^−9^ (1.0 × 10^-9^ - 3.0 ×10^-9^) substitutions per year for cpDNA by Wolfe *et al*. (1987) and 4.76 × 10^-3^ (1.2 × 10^-4^ - 2.7 × 10^-2^) per allele per generation for microsatellite by Cieslarová *et al*. (2011).

**Table S5** Details information of cpDNA primers used in this study.

| Locus | PCR primers (5′ – 3′) | | Ta(℃) | Length | Reference |
| --- | --- | --- | --- | --- | --- |
| *psb*A／*trn*H | *psb*A | GTTATGCATGAACGTAATGCTC | 55 | 504bp | Taberlet *et al*., 1999 |
|  | *trn*H | GTTATGCATGAACGTAATGCTC |  |  |  |
| *trn*L／*trn*F | *trn*L | CGCGCATGGTGGATTCACAATCC | 58 | 1012bp | Sang *et al*., 1997 |
|  | *trn*F | CGAAATCGGTAGACGTACG |  |  |  |

**Table S6** Details information of 10 polymorphic microsatellite loci used in this study.

| Locus | Primer sequence (5′ – 3′) | Repeat motif | Allele size (bp) | Ta (℃) | Fluorescent dye | GenBank Accession No. |
| --- | --- | --- | --- | --- | --- | --- |
| TW01 | F: CTCCAATGCTGCTTCTTTG | (CA)_20_ | 154 – 190 | 56.6 | FAM | JF828274 |
|  | R: AGCCACTTTTCCTAACAATCTG |  |  |  |  |  |
| TW02 | F: CCTTCCTCTTCAGCACAAT | (CT)_29_ | 137 – 172 | 55.7 | FAM | JF828275 |
|  | R: CACAGCCAAATCAAAACAA |  |  |  |  |  |
| TW03 | F: TCAAGCACTTCACCATCCTG | (GA)_19_ | 208 – 240 | 58 | FAM | JF828276 |
|  | R: CGTCCTTTGTTCATTGTGGA |  |  |  |  |  |
| TW04 | F: CCTTTCCATCTCTCCATCTC | (CT)_17_ | 177 – 228 | 56.5 | FAM | JF828277 |
|  | R: GCTTATTTCCAAAGAGTCCAG |  |  |  |  |  |
| TW05 | F: AAGGGTATATTCGTCAATTCAG | (CT)_12_ | 221 – 234 | 56.4 | HEX | JF828278 |
|  | R: GCCAGGATTTTGTATTATAGGA |  |  |  |  |  |
| TW06 | F: TGGTGAAGTGTCCTAATAGAGC | (GA)_10_ | 284 – 294 | 57.3 | HEX | JF828279 |
|  | R: GGTGGCAAGTTCTGTTGTC |  |  |  |  |  |
| TW07 | F: GCGGAGAAATTAAGTTAGGC | (CAT)_10_ (GA)_4_ (AGG)_5_ | 174 – 198 | 56.1 | TAMRA | JF828280 |
|  | R: TGCATGATGACACATACAGAC |  |  |  |  |  |
| TW08 | F: CCGTTGATGTTCTCATAAACTC | (TGA)_8_ | 174 – 201 | 56.2 | TAMRA | JF828281 |
|  | R: TGTTCAGCACTTATTTGACTTG |  |  |  |  |  |
| TW09 | F: ACCAACGGATCAGTTTACATAC | (CAT)_7_ | 196 – 210 | 56.5 | TAMRA | JF828282 |
|  | R: TGGTGGATTTCTAATGATGTTC |  |  |  |  |  |
| TW10 | F: GACGGAGCAGTGTGTAGTG | (CAT)_8_ | 206 – 262 | 56.5 | TAMRA | JF828283 |
|  | R: GAGCAGGAGATTGTGATGAC |  |  |  |  |  |
